# In Vitro Biological Activities of Hesperidin-Related Compounds with Different Solubility

**DOI:** 10.3390/antiox13060727

**Published:** 2024-06-14

**Authors:** Hyo-Jun Lee, Sun-Hyung Lee, Sun-Ki Hong, Bog-Im Gil, Kyung-Ae Lee

**Affiliations:** 1 Graduate School of Biotechnology, College of Life Science, Kyunghee University, Yongin 17104, Republic of Korea; 2 R&D Center, Youngjin Bio Co., Suwon 16614, Republic of Korea; 3 School of Law, Dongguk University, Seoul 04620, Republic of Korea; 4 Department of Food and Nutrition, Anyang University, Anyang 14028, Republic of Korea

**Keywords:** hesperidin, hesperetin, hesperidin glucoside, hesperetin laurate, hydrophobicity, antioxidant, cytotoxicity, anti-inflammatory, antimicrobial

## Abstract

The biological activities of hesperidin-related compounds, such as hesperetin laurate (HTL), hesperetin (HT), hesperidin (HD), and hesperidin glucoside (HDG), were investigated in vitro. The compounds showed different hydrophobicities, and the octanol–water partition coefficient log P were 7.28 ± 0.06 for HTL, 2.59 ± 0.04 for HT, 2.13 ± 0.03 for HD, and −3.45 ± 0.06 for HDG, respectively. In the DPPH assay and β-carotene bleaching assay to determine antioxidant capacity, all compounds tested showed antioxidant activity in a concentration-dependent manner, although to varying degrees. HTL and HT showed similarly high activities compared to HD or HDG. HD and HDG did not show a significant difference despite the difference in solubility between the two. Cytotoxicity was high; in the order of hydrophobicity—HTL > HT > HD > HDL in keratinocyte HaCaT cells. All compounds tested showed reducing effects on cellular inflammatory mediators and cytokines induced by UV irradiation. However, HTL and HT effectively reduced nitric oxide (NO), tumor necrosis factor α (TNF-α), and interleukin-6 (IL-6) levels compared to HD and HDG. The inhibitory effects of hesperidin-related compounds on skin-resident microorganisms were evaluated by measuring minimum inhibitory concentration (MIC). HTL showed the highest inhibitory effects against *Staphylococcus aureus, Cutibacterium acnes, Candida albicans,* and *Malassezia furfur*, followed by HT, while HD and HDF showed little effect. In conclusion, the hydrophobicity of hesperidin-related compounds was estimated to be important for biological activity in vitro, as was the presence or absence of the sugar moiety.

## 1. Introduction

Hesperidin (hesperetin 7-rutinoside, HD) is a polyphenolic secondary metabolite that exists widely in many plants [1]. It is abundantly present in peels of many citrus fruits or peppermints. It is a flavanone glycoside composed of the aglycone hesperetin (HT) and α-L-rhamnosyl-D-glucose (rutinoside). Research on the pharmacokinetics and biological potential of glycoside HD and aglycone HT has been extensively conducted. These studies demonstrate that orally administered HD is generally absorbed in the form of HT after the removal of rutinosides by intestinal microbial hydrolases, and HT undergoes further conversion into active metabolites such as glucuronidated or sulfated HT [2,3]. HD and its derivatives have been reported to possess a wide range of pharmacological properties, including antioxidant, anti-inflammatory, anticancer, antithrombotic, and antimicrobial activities [4,5,6,7,8]. In addition, inhibitory activity against coronavirus and beneficial effects on the skin have also been studied [9,10,11,12].

However, HD and HT have very low solubility in both water and oil, which limits their expanded application in the cosmetics, food, and pharmaceutical industries. Various studies have been conducted to improve this low solubility in both water and oil for biological effectiveness. Improvements in water solubility and antioxidant potential were studied in mixtures of HD and oligosaccharide [13]. Inclusion complexes of HD or HT with various cyclodextrins have been studied to increase water solubility and biological activity [14]. The effect of changing the sugar component in the structure of HD has been extensively studied, and in particular, the glycosylation method has been developed as an efficient way to improve water solubility and bioavailability [15,16,17]. On the other hand, research has been conducted to increase the lipid solubility and efficacy by increasing the hydrophobicity of flavonoids [18,19]. Rutin acylated with unsaturated fatty acids was more efficient than rutin in reducing the secretion of vascular endothelial growth factor from K562 cells, indicating its improved anti-angiogenic properties [20]. Various naringin esters were synthesized enzymatically, and their effects on free radical scavenging activity were studied [21].

**Figure 1:**
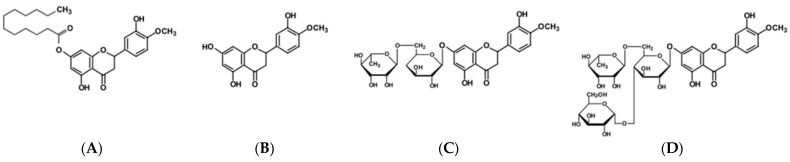
Structure of hesperidin (HD) and its related compounds. (**A**) Hesperetin laurate (HTL), (**B**) hesperetin (HT), (**C**) hesperidin, and (**D**) hesperidin glucoside (HDG).

This study was conducted to investigate and compare the properties and in vitro biological activities of HD and its related compounds, HT, hesperidin glucoside (HDG), and hesperetin laurate (HTL), with different water/oil solubilities. Previously, the properties and efficacy of HT, HD, and water-soluble HDG were compared using antioxidant assays and RAW 264.7 macrophage cells [22]. On the other hand, this study focused on the properties and efficacy via changes in lipophilicity, using lipophilic antioxidant assays and HaCaT keratinocyte cells. The chemical structures of the four compounds used in the study are shown in Figure 1.

## 2. Materials and Methods

### 2.1. Materials and Chemicals

HD (>95%) was obtained from IBT Co. (Gunpo, Republic of Korea). HT and HDG were prepared by the methods described in the previous report [22], and HTL was obtained from Macrocare Co., Ltd. (Ochang, Republic of Korea). For the antioxidant assay, β-carotene and 1,1-Diphenyl-2-picrylhydrazyl (DPPH) were from Sigma-Aldrich Co. (St. Louis, MO, USA). For the cytotoxicity and anti-inflammatory assays, Dulbecco’s modified Eagle’s medium (DMEM) and fetal bovine serum (FBS) were supplied by Hyclone (Logan, UT, USA). The penicillin/streptomycin mixture solution was supplied by Thermo Fisher Scientific Inc. (Waltham, MA, USA), and 3-(4,5-dimethylthiazol-2-yl)-2,5-diphenyltetrazolium bromide (MTT) was purchased from Sigma Chemical Co. (St. Louis, MO, USA). Nitric oxide (NO) was detected using a kit (NO Plus Detection kit^TM^, iNtRON Biotechnology, Seongnam, Republic of Korea). Tumor necrosis factor α (TNF-α) and interleukin-6 (IL-6) were detected using TNF alpha Human ELISA Kit^TM^ and IL-6 Human ELISA Kit^TM^, respectively, which were purchased from Thermo Fisher Scientific Inc. (Waltham, MA, USA). For microbial growth and antimicrobial testing, tryptic soy (TS) broth/agar, yeast malt (YM) agar, Modified Leeming&Notman (MLN) agar and Reinforced Clostridial (RCM) broth/agar were purchased from Kisan Bio Co. (Seoul, Republic of Korea). RPMI-1640 medium and resazurin were from Sigma-Aldrich Co. (St. Louis, MO, USA).

### 2.2. Analysis of HTL

The analytical methods and data of HT, HD, HDG were described in the previous report [22]. HTL was analyzed by the same method as was used for the analysis of HT or HD using an HPLC system (Chromaster 5110, Hitachi, Japan). The column used for HPLC was CAPCELL PAK C18 (UG120 S5, OSAKA SODA, Osaka, Japan). A binary eluent of 0.1% acetic acid (A) and methanol (B) was used as the mobile phase under gradient conditions. The ratio of A:B during elution was 70:30 at 0 min, 0:100 at 5 min, 0:100 at 10 min (0:100), 70:30 at 15 min, and 70:30 at 20 min. The flow rate was maintained at 0.5 mL/min and the column temperature was kept at 30 °C. The detection wavelength for the sample was 280 nm. FT-IR and LC-MS analyses were performed for the instrumental analysis of the HTL structure. The IR spectra of HTL were obtained using an FT-IR Spectrometer (Nicolet iS5, Thermo Fisher Scientific Inc., Waltham, MA, USA), and spectral scanning was performed at wavelengths between 400 and 4000 cm^−1^ with a resolution of 2 cm^−1^. The characteristic absorption patterns were analyzed using the OMNIC 9 software library (Thermo Fisher Scientific Inc., Waltham, MA, USA). The molecular weight of HTL was determined through LC-MS analysis. The Q-TOF Premier Mass Spectrometer (Waters, Milford, MA, USA) was coupled with the HPLC system (Shimazu, Kyoto, Japan), and MS detection was performed in positive ion mode and at a mass range 100~2000 *m*/*z*.

### 2.3. Determination of Partition Coefficient

The hydrophobic/hydrophilic properties were investigated by measuring octanol–water partition coefficients [23,24]. Octanol–water partition coefficients of the four hesperidin-related compounds were determined by the method described in the previous study [22]. Briefly, samples (500 μM) were mixed in deionized water with an equal volume of n-octanol, and then the mixture was kept for 20 h at 20 °C in the dark to reach partition equilibrium. The concentration of each sample was determined by HPLC, and the partition coefficient (log P) was calculated as the ratio of the concentration in the n-octanol phase to the concentration in the water phase.

$$Partition\;coefficient\;Log\left(P\right)=Log\frac{\left[C\right]octanol}{[C]water}$$

where [C]n-octanol is the concentration of a compound in the n-octanol phase, and [C]water is the concentration of a compound in the water phase.

### 2.4. Determination of Antioxidant Capacity

#### 2.4.1. DPPH Assay

Measurements of DPPH radical scavenging activity were performed based on the method described by Ratha et al. [25] and Blois [26], with slight modifications. Briefly, DPPH was dissolved in absolute ethanol to 0.2 mM, and 20 μL of the diluted sample was added to 180 μL of the DPPH solution. The reaction was performed at 37 °C for 30 min to stabilize the color, and the absorbance of the reaction solution was measured at 520 nm using a microplate reader (Epoch2, Bio Tek, Winooski, VT, USA). The DPPH radical scavenging activity was calculated using the following formula. Ascorbic acid was used as a positive control.

$$DPPH\;radical\;scavenging\;activity\left(\%\right)=\frac{A-B}{A}\times 100$$

where A is the absorbance of the reaction solution without a sample, and B is the absorbance of the reaction solution with a sample.

#### 2.4.2. β-Carotene Bleaching Assay

Measurements of the inhibition activity of β-carotene oxidation were performed using the method described by Wanasundara et al. [27] and Hidalgo et al. [28] with slight modifications. Briefly, a mixture of β-carotene and linoleic acid was prepared by dissolving 0.2 mg of β-carotene in 1 mL of chloroform and mixing this solution with 200 mg of Tween 20 containing 20 mg of linoleic acid. After removing the chloroform from the mixture in a rotary evaporator at 50 °C, 100 mL of deionized water was added to prepare an emulsion. The diluted sample (100 μL) was mixed with 900 μL of the prepared emulsion and the absorbance was measured immediately at 470 nm. Then, after a reaction at 50 °C for 2 h, the absorbance was measured again at 470 nm. Antioxidant activity was evaluated as a percent of oxidation inhibition compared to the untreated group sample. As a positive control, BHT (butylated hydroxytoluene) was used in the experiment.

$$\beta -Carotene\;bleaching\;inhibition\;activity\left(\%\right)=\frac{\left(A_{0}-A_{t}\right)-(B_{0}-B_{t})}{A_{0}-A_{t}}\times 100$$

where A_0_ is the initial absorbance without a sample, A_t_ is the final absorbance without a sample, B_0_ is the initial absorbance with a sample, and B_t_ is the final absorbance with a sample.

### 2.5. Determination of Cytotoxicity and Effects on Inflammatory Cytokines

#### 2.5.1. Measurement of Cell Viability

The keratinocyte HaCaT cell line was obtained from AddexBio (SanDiego, CA, USA). The cells were maintained in DMEM supplemented with 10% FBS and 1% of penicillin/streptomycin under a humidified atmosphere condition of 5% CO_2_ at 37 °C. The cytotoxicity of HD and the related compounds was assessed by MTT assay [29]. Cells were seeded in 96-well plates at a density of 2 × 10^4^ cells/well and incubated for 24 h at 37 °C. Each sample of HTL, HT, HD, and HDG was dissolved in DMSO as a stock solution (0.1 M). Different concentrations (12.5–200 μM) of each sample were added to the wells. After incubation for 24 h, the culture medium was removed and MTT solution (100 μL) was added to the well. The mixture was kept at 37 °C for 2 h, then DMSO was added to the reaction mixture and the absorbance at 540 nm was measured in a microplate reader (Epoch2, Bio Tek, Winooski, VT, USA). Cell viability was calculated as a percentage of difference in absorbance between control and test samples.

#### 2.5.2. Measurement of Inflammatory Mediator and Cytokines

Cellular NO levels were determined as an inflammatory intermediate in HaCaT cells. Cells were seeded in 96-well plates at a density of 2 × 10^4^ cells/well and various different concentrations of HTL, HT, HD, and HDG were added and incubated for 1 h at 37 °C. Then, cells with or without samples were irradiate with 10 mJ/cm^2^ of UV rays at 302 nm using a UV irradiator (UV Crosslinker CL-1000M, UVP, Upland, CA, USA). Prior to testing, cell survival against UV damage was found to be over 88% when irradiated with 10 mJ/cm^2^ of UV. After 24 h incubation, the NO content in the media was measured using an NO assay kit according to the manufacturer’s experimental protocol. The absorbance was measured at 540 nm in the same microplate reader as above.

Cellular levels of TNF-α and IL-6 were measured in HaCaT cells after the addition of various concentrations of HTL, HT, HD, and HDG under UV irradiation. The cell culture and sample treatment conditions for the TNF-α and IL-6 assay were the same as those used in the NO assay. The TNF-α and IL-6 contents in the media were measured, respectively, using commercial detection kits according to the manufacturer’s protocols. The absorbance was measured at 450 nm in the same microplate reader as above.

### 2.6. Determination of Antimicrobial Capacity

#### 2.6.1. Microorganisms and Culture Conditions

Antimicrobial capacity was evaluated against some bacteria and yeast known to be major skin resident microorganisms. *Staphylococcus aureus* (KCCM 11335) was cultivated on TS agar, and *Cutibacterium acnes* (KCTC 25526) was cultivated on RCM agar in an anaerobic jar (Rec. Jar, Mitsubishi Gas Chemical Co., Tokyo, Japan) with anaerobic gas packs. *Candida albicans* (KCCM 50235) was cultivated on YM agar, and *Malassezia furfur* (KCTC 7545) was cultivated on MLN agar. All the microbes were cultivated at 35 °C.

#### 2.6.2. Minimum Inhibitory Concentration (MIC)

For the determination of MIC, *S. aureus* was cultivated in TS broth, and *C. acnes* was cultivated in RCM medium in an anaerobic jar with gentle shaking. *C. albicans* was cultivated in an RPMI-1640 medium supplemented with L-glutamine, and *M. furfur* was cultivated in an RPMI medium supplemented with 2% oleic acid, 0.5% glycerol, 0.5% Tween 60, and 0.2% sodium bicarbonate [30,31,32,33]. All the microbes were cultivated at 35 °C, and the inoculum size for MICs was 5 × 10^5^ cells/mL for *S. aureus*, and 1.5 × 10^5^ cells/mL for *C. acnes, C. albicans*, and *M. furfur*, respectively.

MIC values were determined by the broth micro-dilution method [34,35] with some modifications. Briefly, the culture broth of each microorganism was diluted with the same medium. Two-fold serial dilutions of HTL, HT, HD, and HDG ranging from 4000 to 62.5 μg/mL (depending on the microorganism tested) were performed in 96 well plates. After incubation for sufficient growth, the resazurin solution (1 mg/mL, 10 µL) was added to each well and the plate was incubated for 2 h. The MIC value for a microorganism was determined as the lowest concentration of the tested compound that prevented the color change of resazurin (blue to pink).

### 2.7. Statistical Analyses

All the experiments were conducted in triplicate, and the experimental values were represented as mean ± standard deviation. One-way analysis of variance (ANOVA) was performed for comparisons among groups using SPSS software (version 22, SPSS Inc., Chicago, IL, USA), and Duncan’s multiple range test (DMRT) was used to determine significant differences in the treatments at *p* < 0.05 and *p* < 0.01.

## 3. Results

### 3.1. Instrumantal Analysis of HTL

**Figure 2:**
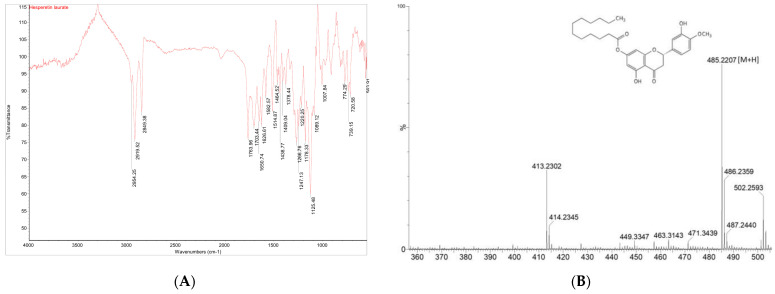
FT-IR and LC-MS spectra of HTL. (**A**) FT-IR spectrum of HTL, (**B**) LC-MS spectrum of HTL.

The HTL used in this study was identified through FT-IR and LC-MS analysis. The characteristic absorption patterns of the ester bond were identified in the 1700~1500 range and 1200~1100 range, as shown in the FT-IR spectra of HTL (Figure 2A). The protonated molecular ion of HTL was identified at *m/z* 485.2207 [M + H] (Figure 2B).

### 3.2. Partition Coefficient of HTL, HT, HD, and HDG

The compounds used in the experiment showed significant differences in terms of water solubility. HTL was extremely insoluble in water, while HDG was highly soluble in water. HT was hardly soluble in water and HD, the rutinose glycoside of HT, was still poorly soluble in water. The solubilities of HTL, HT, HD, and HDG were assessed by measuring the octanol–water partition coefficient expressed in Log P. The Log P value of HTL was the highest, and the value of HT was slightly higher than that of HD, while the value of HDG was much lower than that of HD (Table 1).

### 3.3. Antioxidant Capacity of HTL, HT, HD, and HDG

**Figure 3:**
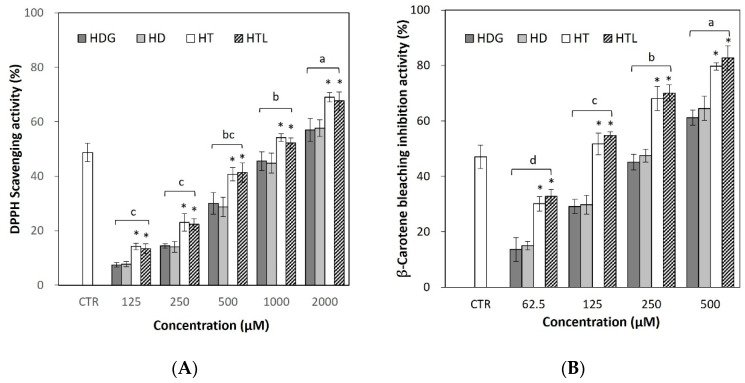
Antioxidant activities of HTL, HT, HD, and HDG. Results are represented as a mean ± SD (n = 3). (**A**) 1,1-diphenyl-2-picrylhydrazyl (DPPH) radical scavenging assay, (**B**) β-carotene bleaching inhibition assay. Different letters (a, b, c, and d) in the column are significant differences according to Duncan’s multiple range test (*p* < 0.05). The symbols * indicate a significant difference (*p* < 0.05) within the group. HTL, hesperetin laurate; HT, hesperetin; HD, hesperidin; HDG, hesperidin glucoside.

Antioxidant capacity was evaluated by measuring DPPH radical scavenging activity and β-carotene bleaching inhibition activity. In the DPPH assay, HTL, HT, HD, and HDG each showed DPPH radical scavenging activity in a concentration-dependent manner (Figure 3A). HTL showed the highest activity among the compounds tested, followed by HT. HT showed a higher effect than HD and HDG, but there was no or little significant difference between HD and HDG. The median inhibitory concentration (IC_50_) representing 50% of radical scavenging was identified as 899.14 ± 2.52 μM for HTL, 893.13 ± 1.67 μM for HT, 1409.41 ± 3.43 μM for HD, and 1407.64 ± 3.73 μM for HDG, respectively. Ascorbic acid, a positive control, showed a scavenging activity of 48.74% at a concentration of 50 μM.

The pattern of results from the β-carotene bleaching assay was similar to that of the DPPH assay, i.e., HTL showed the highest activity, and HT showed a higher effect than HD and HDG (Figure 3B). The IC_50_ of the β-carotene bleaching assay was 105.26 ± 2.01 μM for HTL, 109.71 ± 3.74 μM for HT, 260.75 ± 2.55 μM for HD, and 267 ± 2.69 μM for HDG, respectively. BHT, a positive control, showed an inhibitory activity of 47.08% at a concentration of 25 μM.

### 3.4. Cytotoxicity of HTL, HT, HD, and HDG

**Figure 4:**
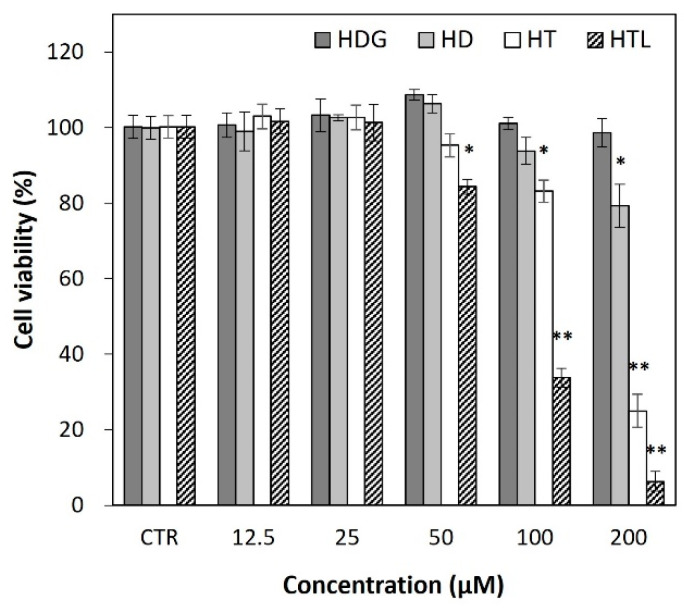
Effects of HTL, HT, HD, and HDG on HaCaT cell viability. Cells were treated with HTL, HT, HD, and HDG, respectively, and incubated for 24 h. The results are represented as a mean ± SD (n = 3). Significant differences from the control (* *p* < 0.05; ** *p* < 0.01). CTR, control (no treatment); HTL, hesperetin laurate; HT, hesperetin; HD, hesperidin; HDG, hesperidin glucoside.

The cytotoxicities of HTL, HT, HD, and HDG were evaluated based on an MTT assay in HaCaT cells. HDG showed much less cytotoxicity than the other compounds, while HTL showed the highest toxicity among them (Figure 4). HDG did not cause significant cell death even at 200 μM, while HD and HT showed cytotoxic effects at 100~200 μM. HTL induced cell death at relatively low concentrations compared to the other samples. Based on these results, anti-inflammatory tests were conducted up to a concentration of 50 μM.

### 3.5. Inhibitory Effects of HTL, HT, HD, and HDG on Inflammatory Mediators and Cytokines

#### 3.5.1. Effects on Cellular NO Levels

**Figure 5:**
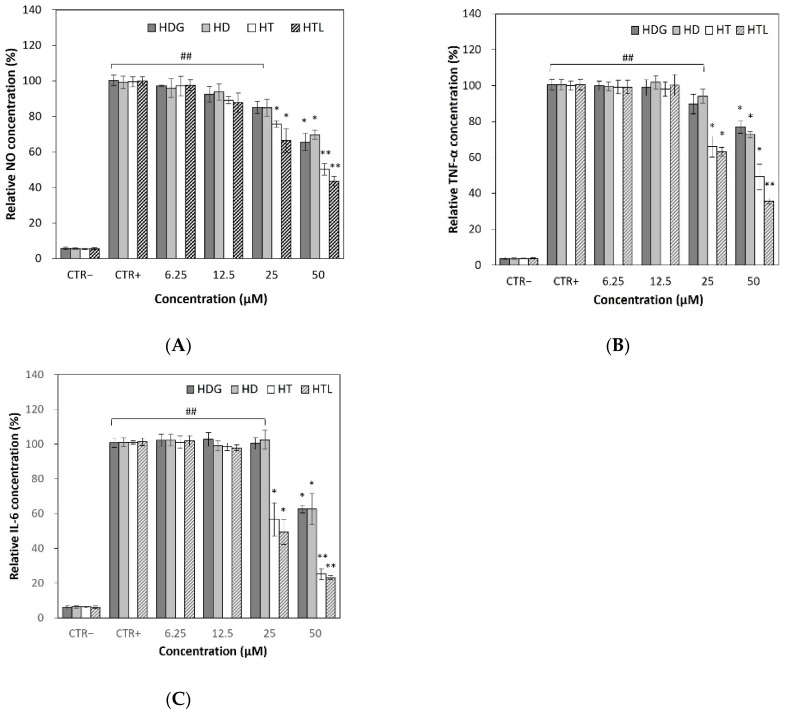
Inhibitory effects of HTL, HT, HD, and HDG on the cellular levels of nitric oxide (NO), tumor necrosis factor (TNF)-α and interleukin (IL)-6. HaCaT cells were cultured and treated with different concentrations of HTL, HT, HD, and HDG for 24 h after UV irradiation. The results are represented as a mean ± SD (n = 3). (**A**) Effect on NO levels; (**B**) effect on TNF-α levels; (**C**) effect on IL-6 levels. The symbol ## indicates a significant difference (*p* < 0.01) compared to CTR−, ** and * indicate significant differences (*p* < 0.01 and *p* < 0.05, respectively) compared to CTR+. CTR−, negative control (no treatment); CTR+, positive control (UV irradiated); HTL, hesperetin laurate; HT, hesperetin; HD, hesperidin; HDG, hesperidin glucoside.

NO is an important inflammatory mediator that can be induced by various forms of chemical or UV irradiation [36,37]. The UV irradiation at 10 mJ/cm^2^ induced significant NO production compared to the non-irradiated control. Treatment with HTL, HT, HD, and HDG suppressed NO production depending on the concentration. NO production was markedly decreased by treatment with HTL and HT at concentrations of 25 μM (Figure 5A), but HD and HDG reduced NO levels at higher concentrations.

#### 3.5.2. Effects on Cellular TNF-α and IL-6 Production

The levels of TNF-α and IL-6, the well-known pro-inflammatory cytokines, were quantified in the culture media of HaCaT cells. The production of TNF-α and IL-6 was induced by UV irradiation, and the application of HTL, HT, HD, and HDG to the cell reduced cytokine production to some extent. Similarly to the NO results, HTL showed the highest reducing effect, followed by HT at a relatively low concentration. Drastic effects were not observed in HD and HDG at tested concentrations up to 25 μM (Figure 5B,C).

### 3.6. Antimicrobial Capacity of HTL, HT, HD, and HDG on Skin-Resident Microorganisms

#### Effects on Microbial Growth

**Figure 6:**
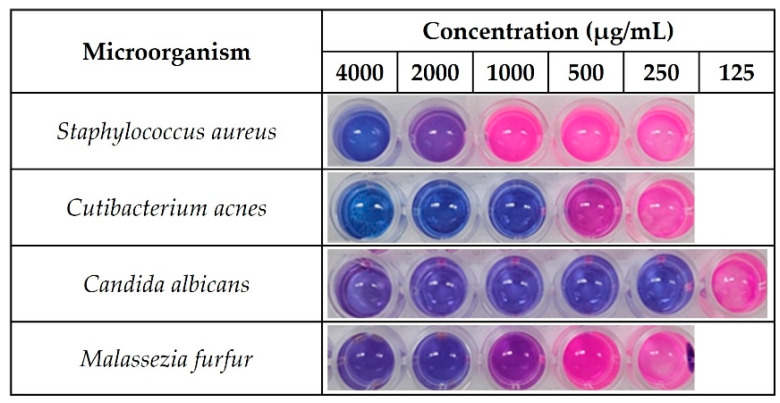
Inhibitory effect of HTL on the microbial growth. Microorganisms were cultivated with different concentrations of HTL.

The antibacterial activities of HTL, HT, HD, and HDG were evaluated by measuring the MICs of *S. aureus, C. acnes, C. albicans,* and *M. furfur*. There were differences in the growth inhibition effects depending on the microbial species, but in most tests, HTL and HT showed a much higher inhibitory effect than HD and HDG. HTL showed the highest inhibitory activity amongst the tested compounds, showing MIC values of 2000 μg/mL against *S. aureus*, 1000 μg/mL against *C. acnes*, 250 μg/mL against *C. albicans,* and 1000 μg/mL against *M. furfur*, respectively (Table 2, Figure 6). HT also showed a relatively higher antimicrobial activity compared to HD or HDG. The test was performed up to 4000 μg/mL, but some of the data at 4000 μg/mL were unclear. Thus, the data were also represented as ND (not detected).

## 4. Discussion

HD is one of the most widely commercially available flavonoids. However, its industrial application has been limited due to its low solubility in both aqueous and oily conditions. It has been demonstrated that changes in the solubility of flavonoids may affect their bioavailability and biological function [38,39]. The conversion of flavonoid glucosides, the storage form in plants, to aglycones, the activated form, has been thought to increase biological activity [40,41]. Studies also show that flavonoid aglycones easily permeate into the intestinal submucosal layer through passive diffusion [42,43]. However, many studies have demonstrated that the increased water solubility of flavonoids may increase their efficacy in vitro and in vivo [44,45]. The glycosylation of HD has been developed as an effective method for increasing the water solubility of HD [46,47]. While much research has been conducted to increase the hydrophilicity of flavonoids, research is also being conducted to improve lipid solubility by increasing hydrophobicity. The chemical modification and enzymatic acylation of flavonoids with fatty acids has been suggested to improve the lipophilicity of the flavonoids [19,48]. Such chemical or enzymatic lipophilization of flavonoids may increase lipid solubility and stability, as well as biological properties. However, there are few reports that directly compare the different effects of hydrophilic and hydrophobic flavonoids simultaneously. In the present study, we investigated the properties and biological effects of hesperidin-related compounds to evaluate the differences between the compounds with different solubilities or hydrophobicities. This research could be utilized to apply the compounds to the non-intestinal systems, such as topical applications to skin or subcutaneous injection.

Flavonoids are generally considered to be potent natural antioxidants, but there are also reports that, depending on the structure, concentration and biological environment, flavonoids can act as pro-oxidants or antioxidants [49,50]. Antioxidant property, which is closely related to many other biological activities including anti-inflammation [51,52], has been investigated prior to determining the anti-inflammatory activity of the hesperidin-related compounds. The effect of the relationship between the structure and activity of flavonoids on their antioxidant capacity has been extensively studied [53]. The antioxidant capacity of flavonoids depends on the location and number of the OH groups, the presence/absence of double bond, 3-OH and 5-OH groups, and the sugar-binding site, etc. It has been reported that the 3′-OH group of flavonoids is important for antioxidant capacity [40]. The aglycone/glycoside structure of flavonoids is important for solubility and antioxidant function, and the antioxidant ability of flavonoids also varies depending on the molecule attached to the aglycone [53].

In this study, a β-carotene bleaching assay was performed in addition to the DPPH radical scavenging assay to test HTL, which is highly lipophilic due to the fatty acid attached. However, as shown in the results, the inhibition patterns between the two assays are similar, except that the IC_50_ values of β-carotene assays are lower than those of the DPPH assay. HTL and HT showed similarly high activity in both the DPPH and β-carotene assays. HTL, derived from the aglycone in HT, and HT showed higher activities than HD and HDG, derived from the glycoside in HD, in both assays. HD did not show a significant difference from HDG despite the differences in solubility. From the test results, it was estimated that structures affecting the hydrophobicity/hydrophilicity had relatively fewer significant effects, and the basic structures of flavonoids, i.e., aglycone and glycoside, had more effects on the antioxidant activity.

Flavonoids have been reported to cause cytotoxicity at certain concentrations in a variety of cells including normal and cancer cells [54,55,56]. However, the mechanism of cytotoxicity has not been clearly understood. It has been suggested that flavonoids may increase intracellular ROS levels, induce apoptosis, and inhibit cellular and mitochondrial membrane permeability depending on cell line and flavonoid type. The cytotoxicity of hesperidin-related compounds was shown in the order HTL > HT > HD ≥ HDG in keratinocyte HaCaT cells. The cytotoxicity was higher in relation to hydrophobicity. It can be assumed that increased hydrophobicity may increase flavonoid–membrane interactions, resulting in increased cytotoxicity [57].

Oxidative stress and resulting inflammation are associated with various diseases, including cancer, neurodegenerative diseases, and cardiovascular diseases [58,59]. Oxidative stress may activate various transcription factors, which can lead to gene expression, including inflammatory cytokines and mediators [60]. Inflammation is one of the more important biological reactions that restore homeostasis in our body. However, excessive inflammation can cause unnecessary damage and have a destructive effect on our body or tissues [61]. Flavonoids have been used as natural anti-inflammatory agents for a long time, and various studies have been conducted to expand their utilization by understanding their properties and pharmacological mechanisms [62]. In this experiment, all tested compounds, HTL, HT, HD, and HDG, showed a concentration-dependent reduction in cellular levels of the inflammatory mediator NO and the inflammatory cytokines TNF-α and IL-6, although there were differences in the degree. HTL showed higher cytotoxicity compared to other hesperidin-related compounds, but on the other hand, it showed high anti-inflammatory effects even at low concentrations. HD and HDG showed relatively low anti-inflammatory effects at the tested concentrations. Comparisons could not be completed at higher concentrations over 50 μM due to different cell viabilities between the compounds tested, but HDG, which has relatively low cytotoxicity, is likely to be effective at higher concentrations.

Many studies have demonstrated that HD and HT exhibit antimicrobial activity [63]. The protective functions of HD and HT against toxicity that may be induced by pathogenic microbes, chemotherapy drugs or UV have also been reported [64,65]. The exact mechanism of the antimicrobial effect has not been fully understood, but some mechanisms have been proposed, including immune system activation, the disruption of membrane permeability, and the inactivation of microbial enzymes [66]. It has been suggested in some studies that increasing the lipophilicity of flavonoids may improve their antimicrobial capacity. Fatty acid esters of flavonoid or aglycone type may be more effective in inhibiting microbial growth than flavonoids of glycoside type [66,67]. In this study, hesperidin-related compounds were tested for their antimicrobial activity against skin-resident microbes, especially the microbes known to be abundant in the oily skin area around sebaceous glands or hair follicles. The excessive growth of the microbes may cause skin inflammation, acne, dandruff, and hair loss under certain conditions [68,69,70]. HTL with high Log P values showed higher antimicrobial activity against microbes tested. The esterified fatty acid may facilitate the antimicrobial action of HT. HT, second to HTL in terms of hydrophobicity, showed high antimicrobial activity following HTL, while HD, with similar Log P values to HT, exhibited much lower antimicrobial activity than HT. Although the lipophilicity of flavonoids is one of the more important properties relating to their antimicrobial activity, as shown in some cases, high lipophilicity is not the only factor that results in high antimicrobial activity [71,72,73]. The relatively large molecular size and hydrophilic sugar moiety of HD may reduce its incorporation into the cell membrane compared to HT. On the contrary, HDG, with a very low Log P value (hydrophilic), showed similarly low antimicrobial activities to HD. It can be assumed that the high solubility of HDG in water facilitates its action in the aqueous medium compared to HD, which has very low water solubility. Considering the molecular weights of the tested compounds (HD, 610.2 Da; HDG, 772.7 Da), HDG can be considered to have higher or at least similar antimicrobial activity compared to HD.

## 5. Conclusions

The biological activities of hesperidin-related compounds with different solubility values (hydrophobicity) were investigated in vitro. HTL and HT showed similarly high activities in antioxidant and anti-inflammatory assays. However, all the hesperidin-related compounds showed antioxidant and anti-inflammatory capacities in a concentration-dependent manner, although there were differences in degree. Cytotoxicity was high in relation to hydrophobicity, and can be ranked as HTL > HT > HD > HDL in keratinocyte HaCaT cells. HTL and HT exhibited potent antimicrobial activities against some skin-resident microorganisms that can be skin-pathogenic under certain conditions. It was estimated that highly hydrophobic hesperidin-related compounds exhibit more potent biological activity than low-hydrophobicity compounds in vitro. However, since all hesperidin-related compounds tested showed biological activity to varying degrees, it is believed that their use can be expanded depending on the properties of the compound. HD has generally been studied to increase hydrophilicity, and has been mainly applied in the food field. The development of highly hydrophobic HD derivatives such as HTL is expected to further expand the range of HD applications. 

## Figures and Tables

**Table 1:** n-Octanol–water partition coefficients of HTL, HT, HD, and HDG.

Compound	Log P_o/w_
Hesperetin laurate Hesperetin	7.08 ± 0.06 ^a^ 2.59 ± 0.04 ^b^
Hesperidin Hesperidin glucoside	2.13 ± 0.03 ^b^ −3.45 ± 0.06 ^c^

The results are represented as a mean ± SD (n = 3). Different letters (^a, b^ and ^c^) in the column denote significant differences according to Duncan’s multiple range test (*p* < 0.05).

**Table 2:** Minimal inhibitory concentration (MIC) of HTL, HT, HD, and HDG.

Microorganism	MIC Value (μg/mL)			
	Hesperetin Laurate	Hesperetin	Hesperidin	Hesperidin Glucoside
*Staphylococcus aureus*	2000	2000	ND	ND
*Cutibacterium acnes*	1000	1000	ND	ND
*Candida albicans*	250	500	2000	2000
*Malassezia furfur*	1000	ND	ND	ND

ND: Not detected.

## Data Availability

Data are contained within the article.
